# Supplementary material for: Investigating the influence of physiologically relevant hydrostatic pressure on CHO cell batch culture
Source: Sci Rep. 2021 Jan 8;11:162. doi: 10.1038/s41598-020-80576-8 (PMC7794228; doi:10.1038/s41598-020-80576-8)
Supplement: Supplementary file 1 — Supplementary Information 1. [file 41598_2020_80576_MOESM1_ESM.zip › S1/IgG/Feb14 0 30 60 90/0mmHg Feb3.pdf]

Data File C:\Users\P...ChemStation\1\Data\TK_CHO_IgG 2019-02-14 10-52-34\012-7-ML 0 Feb3.D
Sample Name: ML 0 Feb3

===

Acq. Operator : SYSTEM Seq. Line : 12
Sample Operator : SYSTEM
Acq. Instrument : HPLC-0039 Location : 7
Injection Date : 2/14/2019 11:41:37 AM Inj : 1
Inj Volume : 100.000 µl
Method : C:\Users\Public\Documents\ChemStation\1\Data\TK_CHO_IgG 2019-02-14 10-52-34 \CHO_IgG_LC.M (Sequence Method)
Last changed : 2/8/2019 3:17:23 PM by SYSTEM

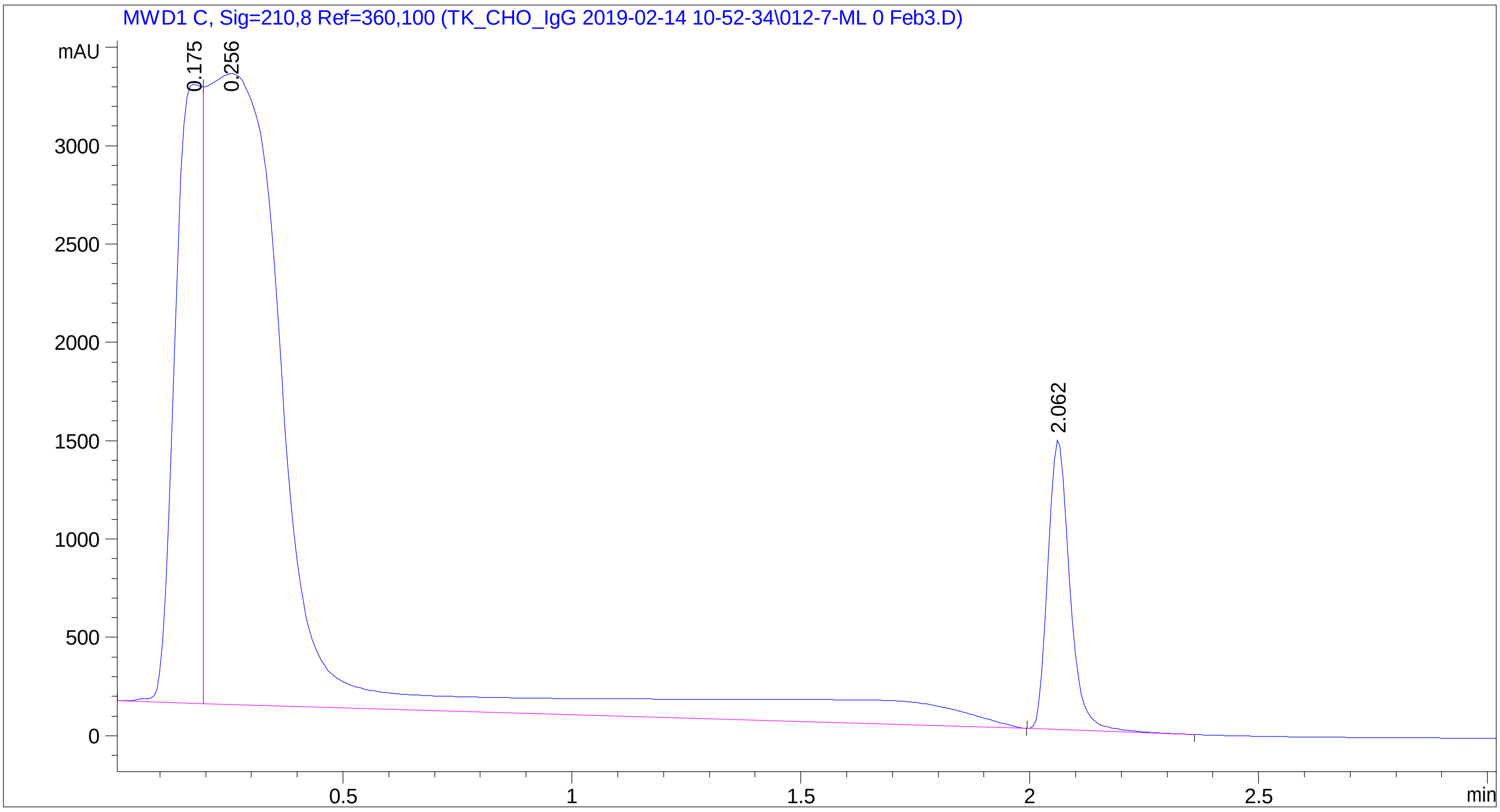

===

Area Percent Report

===

Sorted By : Signal
Multiplier : 1.0000
Dilution : 1.0000
Use Multiplier & Dilution Factor with ISTDs

Signal 1: MWD1 C, Sig=210,8 Ref=360,100

Peak #	RetTime [min]	Type	Width [min]	Area [mAU*s]	Height [mAU]	Area %
1	0.175	BV	0.0633	1.27626e4	3147.86548	21.3016
2	0.256	VB	0.1936	4.22153e4	3206.18604	70.4598
3	2.062	BBA	0.0530	4936.12061	1476.45020	8.2387

Totals : 5.99140e4 7830.50171

===

*** End of Report ***
